# What Prompts Doctors to Recommend COVID-19 Vaccines: Is It a Question of Positive Emotion?

**DOI:** 10.3390/vaccines9060578

**Published:** 2021-06-01

**Authors:** Venerando Rapisarda, Francesca Vella, Caterina Ledda, Massimiliano Barattucci, Tiziana Ramaci

**Affiliations:** 1 Occupational Medicine, Department of Clinical and Experimental Medicine, University of Catania, 95121 Catania, Italy; vrapisarda@unict.it (V.R.); francesca.vella@unikore.it (F.V.); cledda@unict.it (C.L.); 2 Faculty of Human and Social Sciences, University of Enna “Kore”, 94100 Enna, Italy; tiziana.ramaci@unikore.it; 3 Faculty of Psychology, e-Campus University, 22060 Novedrate, Italy

**Keywords:** trust, vaccine, recommendation, locus of control, emotion, work, doctors

## Abstract

Vaccines are among the most successful and cost-effective public health tools and have greatly contributed to eliminating or controlling several serious vaccine-treatable diseases over the past century. To curb the spread of COVID-19, efficacious vaccination is emerging as essential in mitigating the disease and preventing deaths. Health care workers (HCW) are one of the first groups to receive vaccinations, so it is important to consider their attitudes to COVID-19 vaccination to better address barriers to widespread vaccination acceptance. This study aimed to evaluate variables that are linked with the recommendation of vaccines and intention to take-up vaccination against COVID-19 among the HCWs, in the context of the current pandemic. The study was conducted during the first week of the vaccination campaign dedicated to Italian HCWs, beginning in December 2020, and it involved all doctors in a public hospital in Sicily. The following questionnaires were administered: (1) The perceived vaccine trust questionnaire, measuring the degree of trust in vaccines by healthcare professionals both in general and for the protection of healthcare professionals themselves and patients; (2) the positive and negative affect scale-state (PANAS), for assessing positive and negative emotions in relation to their work as “frontline care providers”; (3) The locus of control of behaviour (LCB) to measure the extent to which subjects perceive responsibility for their personal behaviour (internal vs. external); (4) recommendation vaccines item, referring to the intention to recommend vaccination. The findings suggest that socio-demographic control variables (age, gender, and seniority) showed little or no predictive power in vaccine recommendation, while vaccine confidence, positive emotions, and internal locus of control were excellent predictors of vaccine recommendations by doctors. Younger doctors, both in age and experience, are more confident in vaccines and recommend them more frequently. It is essential to improve institutional communication addressed to doctors to enhance their role as vaccination facilitators.

## 1. Introduction

On 31 December 2019, the Wuhan Municipal Health Commission reported a cluster of pneumonia cases of unknown etiology in the town of Wuhan, in Hubei (China), to the World Health Organisation (WHO) [1,2]; on 9 January 2020, the causative agent of this mysterious pneumonia was identified as a novel Coronavirus, and on 11 March 2020, WHO declared the SARS-CoV-2 infection to be a pandemic [3], because of its high transmissibility. Different reports have shown that numerous health care workers (HCW) have contracted COVID-19 in healthcare settings globally [4].

In Italy, there were a total of 2,683,403 infections [5]; infections at work related to SARS-CoV2 and reported to Italian Insurance Against Work Injuries (INAIL) at 31 December 2020, were 131,090. Among these, HCWs are undoubtedly the most affected category, accounting for 38.7% of reports. 79.1% were nurses (80.9% women), followed by 9.2% medical doctors (MD) (48.0% women), 7.3% social assistance operators (85.1% women), and 4.4% non-health service qualified staff (auxiliaries, porters, stretcher bearers) (75% women) [6].

Vaccines are among the most successful and cost-effective public health tools and have greatly contributed to eliminating or controlling several serious vaccine-preventable diseases over the past century [7]. Not only do vaccines prevent the vaccinated individual from developing a potentially serious illness, but they also help protect entire communities by reducing the spread of infectious agents (herd immunity) [7].

To curb the pandemic, in addition to effective public health measures, such as social distancing, wearing face masks, hand washing, avoiding crowded indoor spaces, and educating the general population, efficacious vaccination is emerging as essential in mitigating disease and preventing deaths [8].

HCWs are at greater risk of infection during the COVID-19 pandemic [9] and are one of the first groups to receive vaccinations and to be involved in vaccination operation, so it is important to consider their attitudes about COVID-19 vaccination to better address barriers to widespread vaccination acceptance [10,11].

Vaccinations are considered one of the most important achievements in public health. Despite being one of the most significant public health successes, there has been a contemporary reduction in vaccination coverage and an upsurge in vaccine hesitancy and scepticism, particularly in Western countries [12,13,14,15,16]. The vast majority of those working in the medical field promote the importance of vaccination of individuals [16].

The role of HCWs becomes essential through advice to patients and the community, to improve uptake of vaccinations [16].

In this vein, the aim of this study was to evaluate variables that are linked with the recommendation of COVID-19 vaccines among HCWs, in the context of the current pandemic. The principal aim of the research was to explore variables related to MDs’ trust for vaccines during the very first week of the vaccination national programme. In particular, the research wanted to investigate the role of locus of control, emotions and socio-demographics variables as possible predictors of MDs’ recommendations in the vaccine.

Starting from the above-mentioned observations, we designed a research study with a sample of doctors, during the first week of the national vaccination campaign, measuring the following variables:

Socio-demographic variables (age, gender, and seniority), trust in the vaccine, locus of control, positive and negative emotions, are determinants of recommendation of the vaccination (as an outcome).

## 2. Materials and Methods

### 2.1. Sample

The study was carried out during the first week of the vaccination campaign dedicated to Italian HCWs, beginning in December 2020, that involved all MDs in a public hospital in Sicily (*n* = 459).

The procedure provided for the administration of questionnaires to be carried out during working hours and a questionnaire was given to all doctors in the hospital under analysis, just after they had been vaccinated with the first dose of Pfizer-BioNTech BNT162b2 (BioNTech, Mainz, Germany). It was based on the voluntary participation of MDs and was administered in line with the Helsinki Declaration and Ethics Committee of the University of Catania (approval number 54/2020/PO).

Participants were informed of the main aspects of research and given the chance to refuse to take part and to withdraw at any time. On giving their consent, participants also confirmed that they understood all the instructions and were willing to take part in the research. They then filled in the questionnaires anonymously. Overall, 112 MDs correctly filled in all the sections of their questionnaire (response rate 24.4%). The analysis sample consisted of 40 men and 72 women (64.3%), mostly young adults (average age: 38.8 ± 11.32) and with a low average seniority (mean years = 6.89 ± 5.82). Almost half of the sample, moreover, consisted of interns (*n* = 53, 47%) (see Table 1), with 40% employed in care and screening departments for patients with SARS-CoV-2 (*n* = 45). Overall, more than two-thirds of doctors had signed up for previous vaccination campaigns, for example the flu vaccination, in recent years (*n* = 78, 69.6%).

### 2.2. Measures

The perceived vaccine trust test was developed by HProImmune (Rapporti ISTISAN, 2012) [17,18]. The purpose of this questionnaire is to measure the degree of trust in vaccines of HCW’s both in general and for the protection of HCWs and patients, and identify best practices to improve vaccine take-up rates. According to the reference model (HProImmuni), the questionnaire was developed as a self-report questionnaire, made up of 11 affirmations (items) belonging to a 5-point response scale ranging from 1 (completely disagree) to 5 (completely agree) (e.g., “I believe that vaccines are important for reducing or eliminating serious diseases”, “I believe that vaccination of health workers should be a prerequisite for working in the health sector”); Cronbach’s alpha = 0.87.

The Positive and Negative Affect Scale-State [19,20]. The positive and negative affect schedule (PANAS) is a self-report questionnaire that consists of two 10-item scales to measure both positive and negative affect. Each item is rated on a 5-point scale, from 1 (“not at all”) to 5 (“very much”) (e.g., positive affect: “Excited” “Determined”, negative affect “Guilty”, “Hostile”). Cronbach’s alpha for sub-scale PA = 0.79; Cronbach’s alpha for subscale NA = 0.89.

The Locus of Control of Behaviour (LCB) [21,22] measures the extent to which subjects perceive responsibility for their behaviour (internal vs. external). A 17-item Likert-type scale rating from 0 (completely disagree) to 5 (completely agree) (e.g., “I can anticipate difficulties and take action to avoid them”, “My life is controlled by outside actions and events”). The lower the score, the more internal the locus of control; Cronbach’s Alpha = 0.76.

Recommendation vaccines item refers to the intention to recommend vaccination. The tool is based on a one-item to which one responds through a 3-point Likert scale (0 = no, I do not recommend it; 1 = I sometimes recommend it; 2 = yes, I systematically recommend it).

Socio-Demographic Variables: participants were asked to provide information on socio-demographic characteristics, such as gender, age, and work details, including job title and seniority.

### 2.3. Data Analysis

The research design was correlational and the data were standardised. Differences between measured variables were analysed through independent t-test, whereas the relationships between variables included in the research were analysed through correlations and multiple regressions analysis using SPSS 22 (SPSS Inc., Chicago, IL, USA). Socio-demographic variables were included in the multiple regression at the first step of the analysis, used as control variables.

## 3. Results

From the responses obtained after administering the questionnaire, gender differences arose in relation to trust in the vaccine, emotions and locus of control. Male MDs had more trust in the vaccine than females, more contained emotions—both positive and negative—than women and a more internal locus of control than women (Table 2).

Age is inversely proportional to trust in vaccines (*r* = −0.229, *p* < 0.05) and to their recommendation (*r* = −0.234, *p* < 0.05), as well as seniority (trust in vaccines, *r* = −0.289, *p* < 0.01; recommendation of vaccines, *r* = −0.287, *p* < 0.01). Young doctors, both in terms of age and experience, trust vaccines more and they recommend them more frequently than the others.

The analysis did not detect significant differences between doctors and interns, except for PA (*t* = 2.51, *p* < 0.05), which proved higher in senior doctors than in young interns (mean PA doctors = 3.25, SD = 0.82; mean PA interns = 2.90, SD = 0.66).

No significant differences were found between MDs in close contact with patients affected by SARS CoV-2 and those with no contact with patients affected by SARS CoV-2, except for trust in vaccines (*t* = 2.68, *p* < 0.01). Doctors working in COVID-19 departments (Trust in vaccines = 4.24, SD = 0.46) trust in vaccines more than doctors with no contact with COVID-19 patients (Trust in vaccines = 3.97, SD = 0.57).

Table 3 summarizes the results of the analysis of the correlation between the variables measured in the study. A 2-step multiple regression (using 1000 bootstrap) was conducted to evaluate the predictivity of vaccine recommendation by socio-demographic (Step 1) and measured (Step 2) variables. A low or close to zero level of the predictive power of recommendation of vaccines in control variables was found, whereas trust in vaccines, positive emotions and the locus of control were found to be high predictors of vaccine recommendations (Table 4). Overall, the variables that were considered in this analysis justify the 32% of vaccine recommendation variance.

## 4. Discussion

Italy is among the nations with the lowest levels of vaccine confidence [23]. For example, between February 2017 and January 2018, Italy accounted for 34% of cases of measles occurring in the European Union [23,24,25]_._

This may be related to general scepticism and poor knowledge on the part of HCWs in recommending vaccinations [25,26].

Results observed a positive approach related to vaccines among the HCWs, although the sample was not particularly large. In particular, our results show that a high rate of people (90%) were convinced that the vaccine against SARS-CoV-2 is essential to eliminate (delate) or merely reduce the infection risk. However, there was a level of fear related to this new vaccine, either because it was new and unknown or because of hypothetical and unknown side effects.

The fear of contracting the infection prevailed among workers in COVID-19 departments and also among people that had no direct contact with patients with SARS-CoV-2. It was found that male MDs trust in vaccination more than others, especially true among younger MDs, considering both age and experience. Indeed, they recommend vaccination more frequently than other categories. On the contrary, older subjects, in terms of both age and experience, had less trust in vaccination than the others. Some doubts related to the long-term effect of the SARS-CoV-2 vaccination on health emerged, as did a fear related to lack of knowledge about this.

Trust in vaccines, positive emotions, and locus of control were high predictors of recommendation; some research has shown that age, as a factor capable of affecting perceived risk, inversely influences fear and hesitation in vaccines in healthcare personnel [27,28,29]; however, the results on the present sample confirmed a more positive perception and greater confidence in the vaccines of younger healthcare professionals [30]. Future research will have to clarify exactly the role of age concerning the various variable trusts, recommendations, hesitancy, and fear for vaccine.

Responsibility for public health messaging primarily lies with governments, scientists and medical professionals [31,32,33].

It is increasingly emerging that doctors represent a model for patients and the community. They serve as guides in describing vaccination risks and at the same time, recommending and promoting the importance of mass vaccination, including through awareness campaigns, slogans, or social media networks [33,34].

The literature on other pandemics has clearly shown that perception of risk has a strong cultural component, therefore, communication strategies should be tailored according to the peculiarities of each country [35].

Risk perception and effective response to pandemics can be crucial factors for managing population behaviour, thereby, ensuring the highest compliance to safety measures and norms [36,37,38]_._

Moreover, the efficiency of governments in encouraging preventative behaviour during pandemics is related to population cooperation, which is closely related to risk perception [39,40].

It is crucial that the general population is well aware of the risk involved with vaccination hesitancy. The rise of vaccine hesitancy poses real and existential threats to the prevention and control of vaccine-preventable diseases and will hinder efforts to mitigate the COVID-19 pandemic [41,42]. In the context of a highly-publicised Coronavirus vaccine rollout, initial uptake by HCWs is critical for safety, health system functioning, and public opinion [41,42,43].

Our study aimed to evaluate the propensity of HCWs towards vaccinations. We expected to find considerable confidence and hope in the vaccine, which, in turn, would send an important message to the wider population, in view of the significant psychological influence that HCWs have. This is one of the most important strengths of this study.

The limitation of the study is the small sample size of the group. The results must be considered with extreme caution as a preliminary assessment of a broader research intervention to improve institutional communication addressed to doctors, in order to enhance their role as vaccination facilitators. For more complex designs, including intervention studies and cohort designs, larger samples would be required and would allow for more sophisticated statistical analyzes.

Among the limitations, it should certainly be pointed out that it could have been very useful to include, in parallel with the measurement of the vaccine recommendation, a measure of fear of the potential undesirable effects of vaccines [44]. The next studies, also in order to be able to compare the results, will certainly have to consider other variables already used in the literature [45].

## 5. Conclusions

The questionnaire we used was useful to monitor HCWs’ emotions and attitudes about vaccinations.

The article is the first to contribute to understanding doctors’ vaccination recommendation processes; in particular, its results highlight how the important action of the recommending vaccines by the government passes through that of doctors, and, in particular, in the trust that doctors themselves have in these tools, and the need to underline especially the aspects that reflect positive emotions linked to broad-spectrum vaccination opportunity [46].

Vaccination is an important means to prevent the occurrence and spread of serious infectious diseases and related complications [47]. HCWs have a high risk of exposure and are also the main means of transmission and spread of infections in hospitals, due to their contact with patients [48]. Considering this, HCW vaccinations are the most important in terms of limiting infection and are targeted to protect us, but also, and above all, to prevent transmission to vulnerable people [48,49,50,51]. Although there is much evidence that vaccinations have a fundamental role in the promotion of health safety among HCW, the number vaccinated in developed countries is still below the optimal level. This has led to many health authorities making recommendations on the matter [51,52,53,54].

It is essential to improve institutional communication addressed to doctors to enhance their role as vaccination facilitators [55].

Overall, it is necessary for all parties to work on communicating the message that everyone’s effort is essential to a successful vaccination plan and that behaviour should in line with the emergency nature of the situation [56,57].

In addition, it is essential to create a positive situation based on participation, with less focus on unfavourable negative messages and more work on positive messaging, which improves collaboration among the whole community. Although public health messages that clearly and forcefully dispute myths and falsehoods about vaccine safety and efficacy are essential, challenging false beliefs about vaccination safety and efficacy also risks creating a “backfire effect”, whereby individuals become more entrenched in their false beliefs [58,59].

It is necessary to take simultaneous action on both issues impacting doctors’ confidence in vaccines and on those prevalent in the wider community, particularly given the level of discrimination suffered by HCWs, as they are often associated with COVID-19 risk [60].

Public health institutions should develop and tailor formative and informative campaigns to reduce the main barriers to the immunisation process and promote vaccination take-up among HCWs [31].

## Figures and Tables

**Table 1:** Sample description (*n* = 112 MDs).

Variables	*n*	*%*
**Gender**		
Male	40	35.7%
Female	72	64.3%
Missing cases	0	0
**Professional position**		
Senior doctor	58	52%
Junior doctor	53	47%
Missing cases	1	1%
**Exposed to COVID19**		
Direct contact	45	40%
Indirect contact	67	60%
Missing cases	0	0

**Table 2:** Gender differences among the measured variables.

Psychological Measures	Male (*n* = 40) M (SD)	Female (*n* = 72) M (SD)	*t*	*p*
Trust in vaccines	4.24 (0.52)	3.99 (0.54)	2.32 *	0.05
Positive emotions	3.34 (0.77)	2.98 (0.80)	2.24 *	0.05
Negative emotions	1.88 (0.66)	2.4 (0.94)	−3.24 **	0.01
Locus of control	1.45 (0.53)	1.67 (0.46)	−2.28 *	0.05
Recommends vaccine	1.15 (0.36)	1.22 (0.41)	−0.858	n.s.

n.s. = not significant. * *p* < 0.05. ** *p* < 0.01.

**Table 3:** Descriptive statistics and zero-order correlations among the measured variables.

Psychological Measures	M (SD)	1	2	3	4	5
1. Trust in vaccine	4.07 (0.54)	-				
2. Positive Emotions	3.11 (0.80)	0.308 **	-			
3. Negative Emotions	2.25 (0.90)	−0.129	−0.082	-		
4. LCE	1.60 (0.49)	−0.273 **	−0.193 *	0.521 **	-	
5. Recommends vaccine	1.29 (0.39)	0.500 ***	0.304 **	−46	0.066	-

* *p* < 0.05, ** *p* < 0.01. *** *p* < 0.001.

**Table 4:** Hierarchical regression analyses of socio-demographic factors and measured variables.

Variables	Recommend Vaccines					
	Step 1			Step 2		
	Β	*t*	*p*	Β	*t*	*p*
**Model 1: Main effects of Socio-Demographic variables**						
Age	−0.014	−0.09	n.s.	−0.022	−0.011	n.s.
Gender	0.072	0.77	n.s.	0.077	0.79	n.s.
Seniority	−0.295	−1.86	n.s.	−0.291	−1.80	n.s.
**Model 2: Main effects of Psychological Measures**						
Trust in vaccines				0.511	6.00	0.000
PA				0.211	2.53	0.013
NA				0.007	0.007	0.939
LCB				−0.261	−2.71	0.008
R2	0.077			0.342		
Adj. R2	0.071			0.319		
Omnibus test of regression	F (3, 107) = 3.40 *			F (4, 106) =1 3.75 ***		

* *p* < 0.05. *** *p* < 0.001.
